# Books and Pamphlets Received

**Published:** 1888-11

**Authors:** 


### BOOKS AND PAMPHLETS RECEIVED.

*The Applied Aantomy of the Nervous System*; being a study of this portion of the human body from a standpoint of its general interest and practical utility in diagnosis, designed for use as a text book and a work of reference. By Ambrose L. Ranney, A. M., M. D., Professor of the Anatomy and Physiology of the Nervous System in the New York Post Graduate School and Hospital; Professor of Nervous and Mental diseases in the medical department of the University of Vermont; late adjunct professor of Anatomy and Lecturer on the Diseases of the Genito-Urinary Organs and on Minor Surgery in the Medical Department of the University of the City of New York; late Surgeon to the Northern and Northwestern Dispensaries; Resident Fellow of the New York Academy of Medicine; member of the Medical Society of the County of New York; member of the Neurological Society of New York; author of a "Practical Treatise on Surgical Diagnosis," "Practical Medical Anatomy," "Electricity in Medicine," "The Essentials of Anatomy," etc. Second edition rewritten, enlarged and profusely illustrated. New York. D. Appleton & Co. 1888.

The first edtiion of this work by Dr. Ranney found ready acceptance as a work of ability and merit. In the present book many changes and interesting additions are made. Among these the latest discoveries on the anatomy and physiology of the brain. There are a number of new cuts, and the diagrammatic illustrations constitute an important feature of the book. The present volume is a large book containing about 780 octavo pages. It must prove a most interesting and valuable acquisition to the library of the progressive medical man.

*The Language of Medicine.*—A manual giving the origin, etymology, pronunciation and meaning of the technical terms found in medical literature. By F. R. Campbell, A. M. M. D., Professor of Materia Medica and Therapeutics, Medical Department of Niagara University, New York. D. Appleton & Co. 1888.

The above we regard as a timely and valuable work, especially to medical students, and indeed to medical men who are studious and progressive in their profession. A great aid is here found to the medical student who from defect of early education is unacquainted with Latin and Greek, and who cannot always find in his medical dictionary satisfactory information in regard to the origin, meaning and pronunciation of medcal technicalities. The work contains 4 parts, or leading heads, under each of which are many important subdivisions and details. 1. Origin of the language of medicine. 2. The Latin element in the language of medicine. 3. The Greek element in the language of medicine. 4. Elements derived from the modern languages. Besides a general index referring to the various subjects treated. The work contains an index of words by which any particular word may be found and full information in regard to it be obtained.

*The Dentists' Manual of Special Chemistry.* By Clifford Mitchell, A. B. M. D., Chicago. Published by the author. Sold in Atlanta by Lester & Kurht, No. 7 Whitehall street. Price $1.50.

The work contains 258 pages, and is well written and mostly published in cloth. In it the author claims, and we think truthfully, "to have reduced dental chemistry to a button." He has been engaged in teaching chemistry for ten years and is presumed to know the needs of the student. It certainly contains a great deal of useful and practical information, condensed into a comparatively small space and made plain and easily comprehensible.

*Therapeutics its Principles and Practice* —By H. C. Wood, M. D., L. L. D., Professor of Materia Medica and Therapeutics, and Clinical Professor of Diseases of the Nervous System, in the University of Pennsylvania.

A book on medical agencies, drugs and poisons, with especial reference to the relation between physiology and clinical medicine. The seventh edition. A treatise on Therapeutics enlarged. Rewritten and enlarged. Philadelphia. J. B. Lippincott & Co., London, 10 Henrietta St. 1888.

We have here a work of 900 oc. pages in which we have able information touching the numerous new remedies which have been brought out since the previous edition. Amongst the new remedies which have been carefully treated are " Hydrastin, Strophanthus, Sparteine, Adonidine, Iodol, ichthyol, Paraldehyde, Urethran, Hypnone, Amyline Hydroli, Methylal, Kawa, Papain, Antifebrin, Salol, Bethol, Thallin, etc. Two hundred pages of new matter has been added to the present edition. This is certainly a great and useful work. Giving us the most advanced views of drugs gathered with indefatigable labor from a wide field of literature.

*Disinfection and Disinfectants.*—Their application and use in the prevention and treatment of diseases, and in public and private sanitation. By the Committee on Disinfectants. Appointed by the American Public Health Association, Concord, N. H. 1888.

This is a work of 254 large oc. pages, ably gotten up by a learned committee composed of the following names: Geo. M. Sternberg, M. D., Joseph H. Raymond, M. D., Chas. Smart, M. D., Victor C. Vaughn, M. D., A. R. Leeds, M. D , W. H. Watkins, M. D., Geo. H. Roe, M. D. It is regarded as the ablest work now extant on disinfection and disinfectants.

*Accidents and Emergencies.*—A manual of the treatment of surgical and other injuries in the absence of a physician. By Charles W. Dulles, M. D. Fellow at the College of Physicians of Philadelphia, and of the Academy of Surgery, Surgeon to the out-door department of the hospital of the University of Pennsylvania, and of the Presbyterian Hospital in Philadelphia, etc. Third edition, revised and enlarged, with new illustrations. Philadelphia. P. Blakeston, Son & Co., 1012 Walnut street. 1888. 123 pages.

A very useful little work to both physician and the non-professional reader.

*Physicians' Leisure Library.*—Three separate volumes. One on " Theory and Practice of the Ophthalmoscope," one on " Diseases of the Liver," and one on " Abdominal Surgery." Issued by George S. Davis, publisher, Detroit Mich. He issues monthly a volume on a practical subject by a permanent author.

IN January, 1889, there will be issued from the press of A. L. Chatterton & Co., New York, a new quarterly, entitled the *Journal of Ophthalmology, Otology & Laryngology.*

*Hot Water in the Management of Eye Diseases.*—Some suggestions by Leartus Connor, A. M. M. D., Ophthalmic and Aural Surgeon to Harper Hospital and Detroit Free Children's Hospital. Detroit, 1887.

